# Samurai in Japan: Class System-Related Morphological Differences in Maxillofacial Regions in the Edo Period

**DOI:** 10.3390/ijerph19159182

**Published:** 2022-07-27

**Authors:** Masako Kawada, Yasuhiro Shimizu, Eisaku Kanazawa, Takashi Ono

**Affiliations:** 1 Department of Orthodontic Science, Graduate School of Medical and Dental Sciences, Tokyo Medical and Dental University (TMDU), Tokyo 113-8549, Japan; masako.k.orts@gmail.com (M.K.); t.ono.orts@tmd.ac.jp (T.O.); 2 Department of Anatomy, Nihon University School of Dentistry, Matsudo 271-8587, Japan; kanazawa.eisaku@nihon-u.ac.jp

**Keywords:** maxillofacial morphology, Japanese samurai, 3D scanning, Tokyo, cephalometric analysis

## Abstract

Previous studies have reported that compared to commoners in Japan’s Edo period, samurai had long heads, more dental irregularities, and slightly worn teeth. However, these studies did not measure the mandible or only measured length. Angular analysis is essential to evaluate the maxillofacial morphology, but there are no comparative studies of samurai and commoners. This study explored the differences in maxillofacial morphology between samurai and commoners in the Edo period. Thirty male skeletons (samurai) and thirty-eight male skeletons (commoners) were used as materials from the National Museum of Nature and Science. The selected specimens were adults aged between 20 and 59 years without serious skeletal damage and with stable occlusion of the molars. We used three-dimensional scanning to measure the specimens’ skeletal, alveolar, and facial widths. The mandibular plane angle and the gonial angle were significantly larger in the samurai than in the commoners. The ratio of the intermandibular first molars, interzygomatic arch, and mandibular width was significantly shorter in the samurai than in the commoners. The samurai had a high angle tendency and smaller mandibular width than the commoners, reflecting the class system.

## 1. Introduction

The Japanese society was divided into samurai and commoners by law during the Edo period (AD 1603–AD 1867). In Japan, the ‘bushi’ (warriors) emerged after the 10th century. However, only families who were authorized by the state to engage in military affairs were classified as ‘bushi’. Those who armed themselves privately were not included. In other words, ‘bushi’ was not an occupation but a class in Japan. Moreover, the people who engaged in warfare as their family business were called ‘bushi’.

A samurai was a high-ranking ‘bushi’ who was a vassal of a noble or shogun. There was already a clear division of status between the samurai and the rest of the common people for hundreds of years before the Edo period. In recent years, urban development has progressed in Tokyo, and many burial sites during the Edo period have been excavated. Investigations into early modern archaeology (including the Edo period) have progressed, and correlating the status of burial facilities and remains has become possible. The physical characteristics of old Japanese people, who were classified in the Edo period as those with different statuses, have also been compared and analyzed [1,2,3].

Compared to commoners, the samurai class had a lower incidence of caries but more tooth wear, probably owing to brushing [4,5,6]. It is also known that the samurai class had a long head, delicate skull and mandible, a high rate of crowding, and a small amount of attrition [1,7]. In contrast, interracial comparative studies using cephalometric analysis have been actively conducted, and several studies have examined skeletal differences between Caucasians and the Japanese [8,9,10,11]. Based on these reports, the Japanese have more protruding mandibular incisors and protruded lip positions than Caucasians.

Some studies compared the heights of lower- and upper-class youth in England in the late 18th and early 19th centuries with those in Europe and North America in the same period, respectively [12]. During that time, the gap between the rich and poor in England was so notable that the height difference reached 22 cm in those aged 16 years. England’s poorest youth were among the shortest in Europe and North America during that time. Moreover, in a study of long-term trends in socioeconomic differences in height among young adult males in Sweden in the 19th and 20th centuries, fathers with white-collar occupations were consistently taller than others at all ages [13]. Studies have compared the whole body, which is highly affected by dietary habits. In addition, a study compared the morphological changes of the mandible from the pre-industrial Middle Ages to the post-industrial early modern period in London [14]. According to this study [14], the post-industrial group of both men and women had a smaller gonial angle, ramus height, and ramus width and a shorter distance between gonions than the pre-industrial group. The authors suggested that this could be due to differences in diet, as food processing technology advanced with the Industrial Revolution, and many people no longer made or grew their own food but purchased conveniently processed foods.

However, a study in Japan compared women in the Edo and modern periods using orthodontic parameters [15]. The study used a standard lateral cephalometric radiograph, and women in the Edo period had flatter occlusal plane angles and bimaxillary dentoalveolar protrusion than those in the modern period [15]. However, there have been no comparative studies between samurai and commoners in the same period of the Edo era. The purpose of this study was to evaluate the morphological differences in the maxillofacial region between two socially different classes—the samurai and commoners—from an orthodontic perspective. The use of three-dimensional (3D) scanning systems in this study aimed to overcome the methodological disadvantages of the methods adopted in previous studies.

## 2. Materials and Methods

The materials used in this study were Japanese skeletal remains that were excavated from three historic sites—Ikenohata, Sugenji Temple, and Syokenji Temple—located in Tokyo. These sites were in operation during the late 17th to 19th century. The remains were stored at the National Museum of Nature and Science, Japan. We were allowed to use the materials as researchers at the National Museum of Nature and Science. Samurai and commoners were buried in coffins made of ceramics and wood, respectively [3,15]. A total of 30 male skeletal remains from ceramic coffins and 38 male skeletal remains from wooden coffins were included in the study. According to a previous study, these materials satisfied the following criteria: (1) men between the ages of 20 and 59 years, (2) absence of severe damage to the skeletal remains, and (3) stable occlusion in the molar region. Estimates of the age and sex of the skeletal remains were obtained from previous reports [1,2].

**Figure 1:**
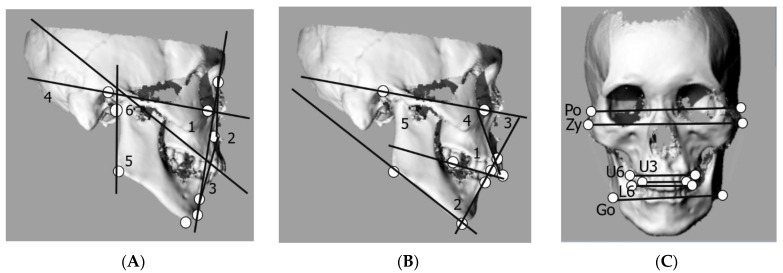
Measurement variables. (**A**) Skeletal angular measurements: 1, facial angle; 2, angle of convexity; 3, A-B plane angle; 4, mandibular plane angle; 5, gonial angle, 6; ramus plane angle to the Frankfort horizontal (FH) plane. (**B**) Dentoalveolar angular measurements: 1, interincisal angle; 2, L1 to mandibular plane angle; 3, Frankfort-mandibular incisor angle; 4, U1 to the FH angle; 5, occlusal plane angle to the FH plane. (**C**) Linear measurements related to the width of the jawbones, width between the porions, width between the upper canines, width between the upper first molars, width between the lower first molars, width of the zygomatic arch, and width of the mandible.

Following previous studies, the maxillary and maxillary dentition were positioned in centric occlusion [16,17]. In that state, the skeletons were 3D-scanned using a porTable 3D scanner (Artek Eva, New York, NY, USA). The 3D data obtained were analyzed using 3D modeling software (Rhinoceros 5.0, New York, NY, USA). The following anatomical landmarks were used as measurement points: porion (Po), orbitale (Or), nasion (Na), point A (A), point B (B), pogonion (Pog), menton (Me), gonion (Go), and articulare (Ar) (Table 1). In the lateral view, the following six angular measurements were computed for skeletal hard tissue analyses: facial angle, angle of convexity, A-B plane angle, mandibular plane angle, gonial angle, and ramus plane angle to the Frankfort horizontal (FH) plane (Figure 1A). Similarly, the following five angular measurements were computed for dental hard tissue analyses: interincisal angle, L1 to the mandibular plane angle, Frankfort-mandibular incisor angle (FMIA), U1 to the FH angle, and occlusal plane angle to the FH plane (Figure 1B). As most of the roots on the skeletal remains were not exposed, the line connecting the center of the incisal edge and the center of the buccal cervical region was used as the tooth axis, as described in a previous study [16]. However, in the frontal view, the following widths were measured: between the bilateral Po, between the incisal tips of the canines on both sides of the maxilla, between the maxillary first molars, between the mandibular first molars, the zygomatic arch, and the mandible (Figure 1C). The widths were converted to values relative to the length between the Po to capture them as a face ratio, as described in a previous study [18].

Landmark identification errors were estimated by examining the 3D data of 20 randomly selected skeletal remains 2 weeks after the first scan. For the test of error in the landmark identification of angle and length measurements, Dahlberg’s formula was used to examine the reproducibility of the measurement. Since it was within the acceptable range, the average of the two measurements was used as the representative value. To compare the mean values of the parameters between the samurai and commoner groups, we first tested if the sample data had equal variances. If Levene’s test showed *p* > 0.05, we performed the unpaired t-test; otherwise, we performed the Welch test. All statistical analyses were performed using SPSS (version 25; IBM, Armonk, North Castle, NY, USA). Statistical significance was set at *p* < 0.05. This study was approved by the Committee of Tokyo Medical and Dental University (approval number 03-2773).

## 3. Results

**Figure 2:**
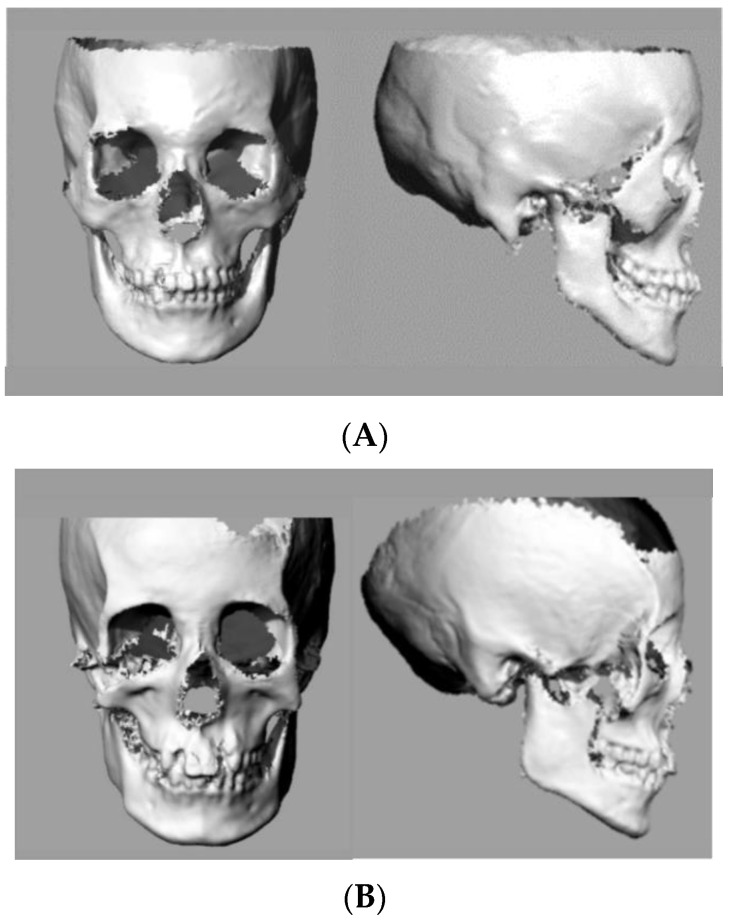
Representative 3D images: (**A**) samurai; (**B**) commoner.

**Figure 3:**
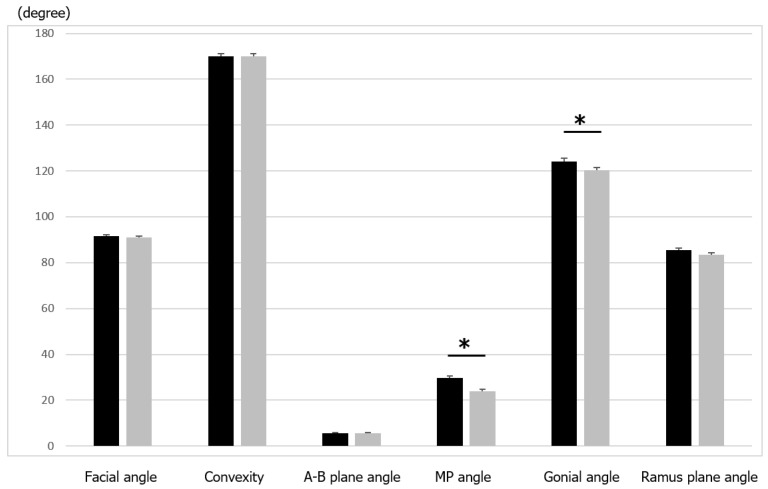
Comparison of skeletal variables between samurai and commoners. Values are presented as means ± standard deviations. The asterisks represent significant differences between the groups. Black, samurai group; grey, commoners.

Representative 3D images of a samurai and a commoner are shown in Figure 2A,B, respectively. In the comparison of skeletal variables, the mandibular plane angle and the gonial angle were significantly (*p* < 0.05) larger in the samurai group than in the commoner group (Table 2 and Figure 3). There were no significant differences in the facial angle, angle of convexity, A-B plane angle, and ramus plane angle to the FH plane. These data indicate there were significant morphological differences in the mandible between the samurai and commoners.

**Figure 4:**
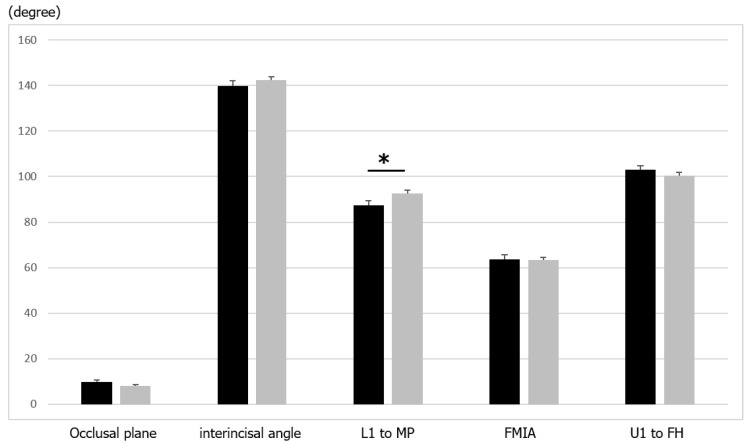
Comparison of dentoalveolar variables between samurai and commoners. Values are presented as means ± standard deviations. The asterisks represent significant differences between the groups. Black, samurai group; grey, commoners.

In the comparison of alveolar variables, L1 to the mandibular plane was significantly (*p* < 0.05) smaller in the samurai group than in the commoner group (Table 3 and Figure 4), which indicated the lower incisor was more upright in the samurai group There were no significant differences in the interincisal angle, FMIA, U1 to the FH angle, and occlusal plane angle to the FH plane.

**Figure 5:**
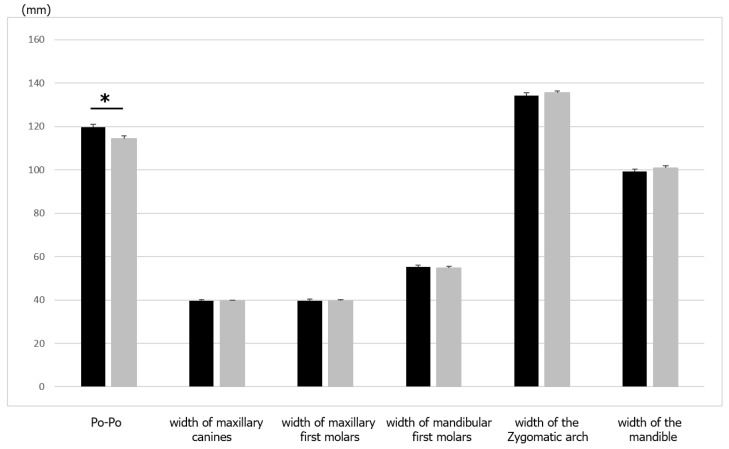
Comparison of the maxillary and mandibular width between samurai and commoners. Values are presented as means ± standard deviations. The asterisks represent significant differences between the groups. Black, samurai group; grey, commoners.

**Figure 6:**
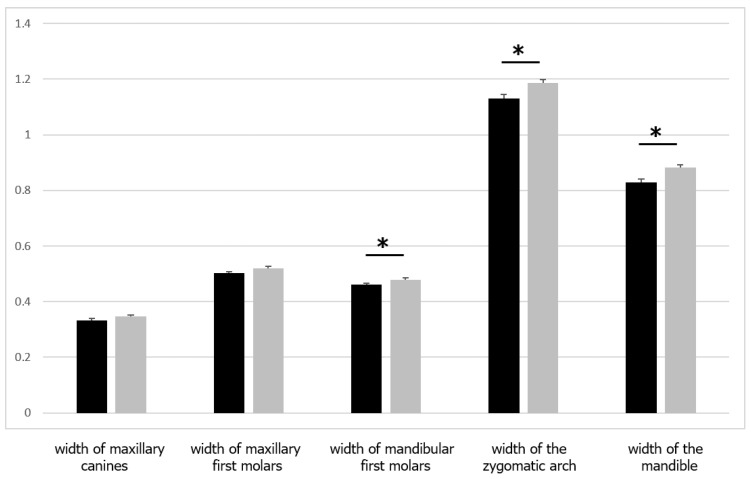
Comparison of the maxillary and mandibular width ratio between samurai and commoners. Values are presented as means ± standard deviations. The asterisks represent significant differences between the groups. Black, samurai group; grey, commoners.

In the comparison of maxillary and mandibular widths, the measured width between the porions was significantly (*p* < 0.05) larger in the samurai group than in the commoner group (Table 4 and Figure 5). However, the ratio of the intermandibular first molars, interzygomatic arch, and mandibular width was significantly larger (*p* < 0.05) in the commoner group than in the samurai group (Table 5 and Figure 6). No significant differences were observed in the widths of the intermaxillary canines and first molars between the two groups. These data indicate that there were no differences in the width of the maxillary teeth between the samurai and commoner groups, but differences were observed in the width of the mandibular and zygomatic bones, where the masseter muscle attaches.

## 4. Discussion

This study aimed to elucidate the skeletal differences between samurai and commoners during the Edo period from an orthodontic perspective. Previous studies reported that among samurai and commoners, samurai had longer heads, more dental irregularities, and a lower amount of tooth wear. However, most of these studies used measurement methodologies that did not include the mandible, or they used points that differed from orthodontic measurement points. In addition, the measurement methods only involved the use of calipers or two-dimensional measurements using radiographs or photographs of the specimen. Previous studies have used two-dimensional (2D) radiographic images [1,6,7], and to the best of our knowledge, no study has scanned specimens in 3D. Analysis using 2D images can affect the results depending on the angle from which the specimen is taken and how the specimen is positioned. Positioning is highly important in studies that measure angles and lengths; thus, 3D images seem more accurate and reliable than 2D. This is the first study to use 3D scanners to compare the maxillofacial morphologies of samurai and commoners using orthodontic measurement points. Interestingly, the results showed that samurai, who supposedly engaged in warfare as their family business, had a higher mandibular plane angle and a smaller mandibular width than commoners.

Historically, samurai first emerged in Japan in the 10th century. However, it was later in the 15th and 16th centuries that the term samurai referred to those who watched over and served noblemen. A samurai was a person who lived by the sword and was not necessarily an officer. It was not a profession of choice but a privileged class. There was no back and forth between the ranks. By the Edo period, the samurai had been around for more than 100 years and had become an established status. In addition, the Edo period was a period of national seclusion owing to policies that cut off foreign exchange. The skull specimens used in this study were all unearthed in what is now Tokyo since it was the center of the capital during the Edo period, and there were more samurai living there than in any other area. This study focused on the Edo period rather than other periods because it was the most mature period (more than 100 years after the emergence of the samurai).

It has been reported that morphological differences exist in the mandible of samurai and commoners and that samurai have more crowded dentition than commoners [1,7]. In this study, the mandibular plane and gonial angles were significantly larger, and the ratio of the mandibular width was significantly smaller in the samurai. According to a previous study [2], the rate of absent third molars, which is influenced by genetic factors, body size, and the environment at the time of childbirth, was higher in the samurai class than in the common class. There were social and cultural differences as well as differences in eating habits and cooking methods between social classes in the Edo period [19,20]. There are records that show that the number of meals and meal contents of the samurai class were restricted. A study that examined the skeletal and dental morphology of the upper-class samurai during the Edo period reported that they had significantly less tooth wear than common people, suggesting that their lifestyle, including their diet, was unique for generations [21]. In the early Edo period, the samurai ate white rice as their staple food, while the commoners ate mixed millet or Japanese millet with white rice. Millet and Japanese millet were harder than white rice and required more effort to grind thoroughly. Compared to common people, samurai were eating different diets for generations, which may explain the differences in mandibular morphology.

In addition, many animal experiments have shown that differences in the shape of the food affect the growth and function of the masticatory muscle, which ultimately affects the growth of the mandible [22,23]. We also investigated the relationship between occlusion and mandibular morphology. We conducted animal experiments using rats with reduced occlusal function in the molar region by attaching appliances to their maxillary and mandibular incisors and clarified the histological changes in the alveolar bone of the mandible. Our findings showed a close relationship between the decrease in occlusal function in the molars and alveolar bone loss in the mandible [24,25]. Another study demonstrated that the mandible shortens in an anteroposterior direction when mice consume a soft diet over multiple generations [26]. Sakaue compared the changes in maxillofacial morphology during the Edo period and reported that the differences between the samurai and commoners were more pronounced during the middle-to-late Edo period. It is thought that skeletal changes depending on status were accumulated, and that such traits were more likely to have appeared in the middle-to-late Edo period [17]. The results of this study showed that the samurai had a larger mandibular plane angle and gonial angle than the commoners. This result may be attributed to the fact that the change in mandibular morphology due to the multigenerational difference in diet between the samurai and commoners was more pronounced in the Edo period when hundreds of years had already passed since the appearance of the samurai.

With regard to arch width, the maxillary canine width and first molar interdental width were not significantly different between the two groups; however, the interzygomatic arch, intermandibular first molar width, and mandibular width were significantly smaller in the samurai class. The commoners generally had more opportunities to eat harder food than the samurai, so it was expected that there would be more tooth wear and the mandibular molars would be more upright buccally [18,20]. Furthermore, we speculate that bone apposition may have occurred in the zygomatic arch and mandibular angle area due to augmentation by hard food in the commoners’ masseter muscle, the main jaw-closing muscle that originates in the zygomatic arch and stops at the masseter muscle rough surface in the mandibular angle [27]. The widths of the zygomatic arch and mandible, which are the attachment sites of the masseter muscles, and the width of the mandibular first molars were significantly smaller in the samurai than in the common people. In other words, it can be assumed that the samurai had a lower bite force than the common people, making it more difficult for the mandibular molars to stand upright buccally. A previous study showed that the mandibular molars of long-faced subjects were more lingually inclined than those of short-faced subjects [28]. Another interesting study reported that Fijians with strong occlusal forces had wider mandibular arch diameters than the Japanese [29]. They compared the data collected from Fijian and Japanese dental casts, cephalometric radiographs, and thin pressure-sensitive sheets for bite force analysis. The occlusal contact areas of the Fijians were also greater than those of the Japanese participants. Fijians also had longer palates, mandibles, and greater bimaxillary protrusion.

In a study comparing modern and ancient Japanese from around BC 5000 to BC 300, long before the Edo period, tooth wear was significantly greater and buccolingual tooth inclination was more vertical in ancient people who probably applied stronger masticatory forces than modern people. Furthermore, those who performed grinding-type masticatory movement had a larger bite force, and their lower molars were more upright buccally [30,31]. Compared to the commoners, the samurai had a smaller mandibular first molar width, which suggests that their occlusal force was weaker, and the width of their masticatory path was smaller.

In another animal study [32], we divided growing rats into control and soft diet groups and fed each group for 9 weeks to analyze the 3D microstructure of the alveolar bone of the first molar region using microtomography. The results showed that the bone marrow space of the alveolar bone in the mandibular first molar region was higher in the soft diet group than in the control group, suggesting that alveolar bone loss was more extensive in the mandible than in the maxilla. In the present study, there was no difference in the maxillary width ratio between the samurai and commoners; however, there was a significant difference in the mandibular width ratio and the ratio of the width between the first mandibular molars.

Our results showed the difference in the maxillofacial skeleton between the different social classes in the Edo period, supporting the results of previous reports by anthropologists from an orthodontic perspective.

## 5. Conclusions

Our study found that samurai had the following morphological characteristics: larger mandibular plane angle and smaller mandibular width compared to commoners. This could be attributed to the differences in diet and eating habits between samurai and commoners for more than one hundred years.

## Figures and Tables

**Table 1:** Landmarks for the 3D analysis.

Landmarks	Interpretation
Porion (Po)	The most lateral point on the roof of the bony external acoustic meatus, vertically over the middle of the meatus
Orbitale (Or)	The lowest point of the infraorbital margin
Nasion (Na)	The point on the skull corresponding to the middle of the nasofrontal suture
Point A (A)	The most posterior midline point on the premaxilla between the anterior nasal spine and prosthion
Point B (B)	The most posterior midline point, above the chin and on the mandible between infradentale and pogonion
Pogonion (Pog)	The most anterior point of the chin on the mandible in the midline
Menton (Me)	The most inferior point on the chin in the lateral view
Gonion (Go)	A point at the intersection of lines tangent to the posterior border of the ramus and the lower border of the mandible
Articulare (Ar)	A point at the intersection of the image of the posterior margin of the ramus and the outer margin of the cranial base
U1	Axis of the maxillary central incisor constructed between the tip of the crown and apex
L1	Axis of the mandibular central incisor constructed between the tip of the crown and apex

**Table 2:** Comparison of skeletal variables between samurai and commoners.

Variables (Degrees)	Samurai	Commoners	Probability
Facial angle	91.49 ± 3.67	91.03 ± 3.44	NS
Angle of convexity	169.87 ± 6.79	170.09 ± 7.22	NS
A-B plane angle	5.55 ± 2.49	5.53 ± 2.83	NS
Mandibular plane angle	29.59 ± 5.27	23.74 ± 5.72	*
Gonial angle	124.15 ± 6.75	120.09 ± 6.86	*
Ramus plane to FH	85.35 ± 5.21	83.53 ± 5.03	NS

Values are presented as means ± standard deviations. * *p* < 0.05. FH, Frankfort horizontal. NS, not significant.

**Table 3:** Comparison of dentoalveolar variables between samurai and commoners.

Variables (Degrees)	Samurai	Commoners	Probability
Interincisal angle	139.77 ± 11.41	142.39 ± 7.38	NS
L1 to mandibular plane	87.10 ± 11.20	92.56 ± 7.28	*
FMIA	63.78 ± 9.56	63.27 ± 6.54	NS
U1 to FH	102.87 ± 8.68	100.48 ± 7.12	NS
Occlusal plane to FH	9.80 ± 4.91	8.06 ± 4.35	NS

Values are presented as means ± standard deviations. * *p* < 0.05. FMIA, Frankfort-mandibular incisor angle; FH, Frankfort horizontal. NS, not significant.

**Table 4:** Comparison of maxillary and mandibular width between samurai and commoners.

Length (mm)	Samurai	Commoners	Probability
Po-Po	119.57 ± 6.98	114.49 ± 6.45	*
Intermaxillary canines	39.62 ± 2.26	39.59 ± 2.02	NS
Intermaxillary first molars	59.54 ± 2.80	59.32 ± 3.17	NS
Intermandibular first molars	55.14 ± 3.41	54.85 ± 3.31	NS
Interzygomatic arch	134.33 ± 5.66	135.64 ± 4.40	NS
Mandibular width	99.21 ± 5.45	100.91 ± 6.18	*

Values are presented as means ± standard deviations. * *p* < 0.05. NS, not significant.

**Table 5:** Comparison of the maxillary and mandibular width ratio between samurai and commoners.

Ratio	Samurai	Commoners	Probability
Intermaxillary canines	0.33 ± 0.03	0.35 ± 0.03	NS
Intermaxillary first molars	0.50 ± 0.03	0.52 ± 0.04	NS
Intermandibular first molars	0.46 ± 0.02	0.48 ± 0.04	*
Interzygomatic arch	1.13 ± 0.07	1.19 ± 0.07	*
Mandibular width	0.83 ± 0.05	0.88 ± 0.07	*

Values are presented as means ± standard deviations. * *p* < 0.05. NS, not significant.

## Data Availability

The data presented in this study are contained within this article.
